# Surgical Correction of a Complete, Symmetric, Longitudinal Vaginal Septum Using LigaSure Technology: A Case Study

**DOI:** 10.7759/cureus.109200

**Published:** 2026-05-19

**Authors:** Kehui Teoh, Carlos Garcia-Jasso, Peggy Taylor

**Affiliations:** 1 Obstetrics and Gynecology, Sam Houston State University College of Osteopathic Medicine, Conroe, USA

**Keywords:** ligasure, longitudinal vaginal septum, müllerian duct anomalies, surgical resection, uterine didelphys

## Abstract

A longitudinal vaginal septum is a congenital anomaly resulting from incomplete Müllerian duct development and is often associated with uterine didelphys, which can lead to reproductive symptoms and challenges. Surgical correction may be necessary for symptomatic individuals, those facing reproductive barriers, or women planning for vaginal birth. We present the case of an 18-year-old woman with uterine didelphys and a complete symmetric longitudinal vaginal septum who elected surgical resection of the septum. The procedure was performed using a LigaSure device and was uncomplicated. This case highlights a successful surgical outcome and supports the LigaSure device as a promising option for the management of longitudinal vaginal septa.

## Introduction

A longitudinal vaginal septum is a rare congenital reproductive anomaly that arises from incomplete fusion or reabsorption of the Müllerian ducts [1]. Specifically, this condition results from defective lateral fusion or a failure to fully resorb the caudal-central portion of these ducts during embryogenesis [1]. In clinical practice, this condition frequently co-occurs with uterine didelphys, characterized by two separate uteruses, endometrial cavities, and cervices, due to Müllerian duct agenesis [1]. While a rare congenital anomaly, longitudinal vaginal septa are anatomically diverse and can be described by their extent (completeness), symmetry, relationship to the cervix, and involvement of the vaginal introitus [2]. The true incidence of such congenital Müllerian defects may be significantly understated due to the absence of symptoms and the prevalence of undiagnosed anomalies in the general population [1]. However, when symptomatic, women with such Müllerian anomalies may face reproductive challenges, including dyspareunia, infertility, difficulties with tampon use, and persistent vaginal bleeding, despite tampon insertion [1,2]. Surgical intervention may be considered to alleviate symptoms, overcome reproductive barriers, or in anticipation of vaginal delivery. Here, we describe a case where the LigaSure device was employed to achieve successful resection of a longitudinal vaginal septum in a reproductive-aged woman.

## Case presentation

An 18-year-old woman presented to her obstetrics and gynecology clinic for elective resection of a longitudinal vaginal septum. Several months prior, she was diagnosed with uterine didelphys and a complete symmetric longitudinal vaginal septum during her first pregnancy. Her pregnancy was complicated by intrauterine fetal growth restriction, as the fetus resided in her left uterus. She underwent an uncomplicated cesarean section, ensuring maternal and fetal safety. She recovered well after the cesarean section and was discharged on postpartum day two. The patient’s past medical, surgical, and family histories were unremarkable. Her obstetrical history was significant only for the previously mentioned pregnancy (G1P1001), with no documented gynecological history of irregular menses, pelvic infections, or prior reproductive tract surgeries. 

When presenting for her well-woman exam, the patient reported dyspareunia and mechanical difficulty with intercourse attributed to the known vaginal septum. Notably, these symptoms culminated in a traumatic tissue tear of the distal septum during sexual activity, which prompted her to seek definitive surgical resection of the remaining proximal segment, near the cervices.

**Figure 1:**
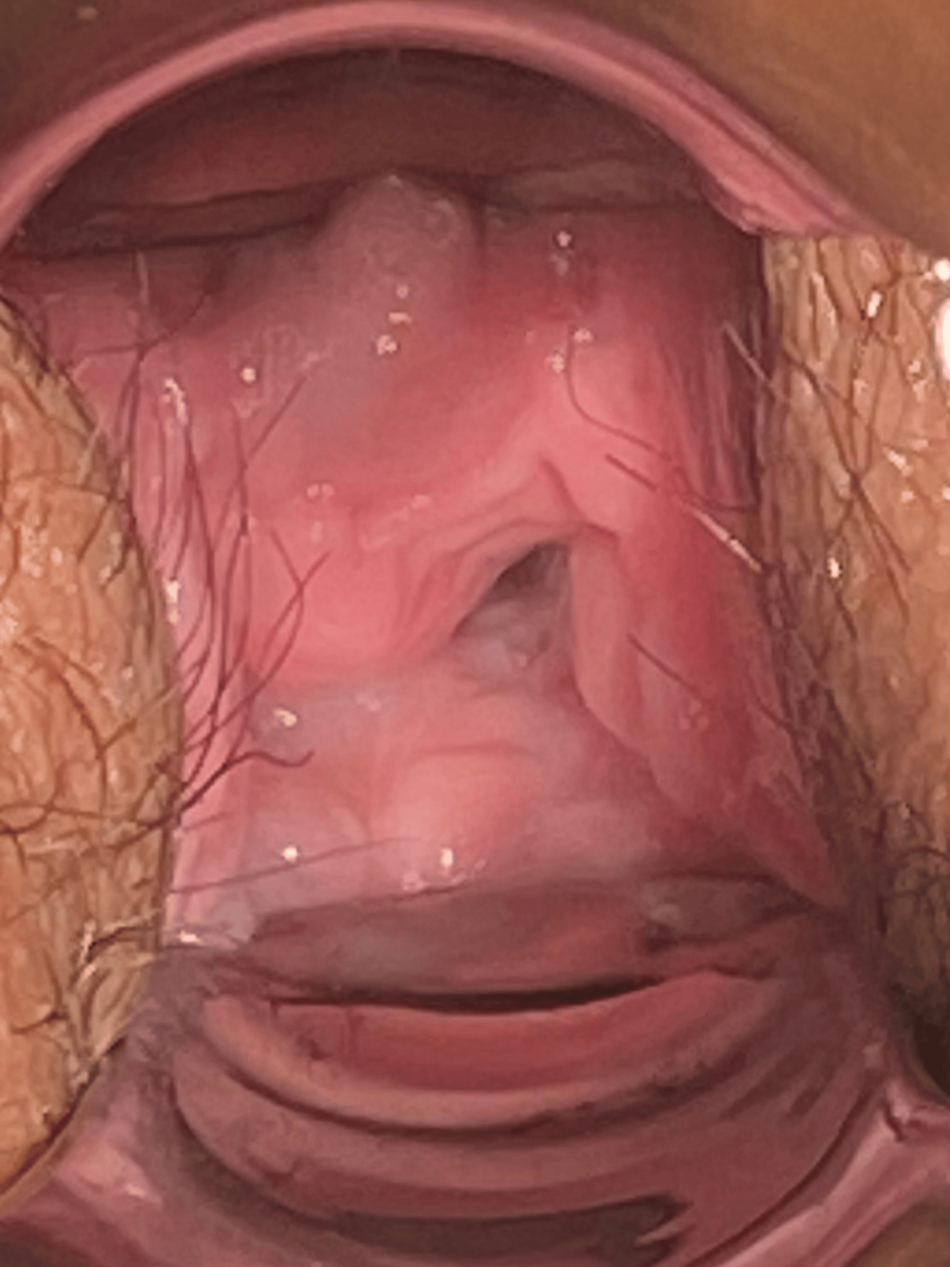
Postoperative appearance following surgical resection of a longitudinal vaginal septum

On the day of surgery, informed consent was obtained and the patient was transferred to the operating room. General anesthesia and prophylactic antibiotics were administered and the patient was placed in the dorsal lithotomy position. Standard sterile preparation and draping were performed. A Foley catheter was inserted for bladder drainage and intraoperative urethral identification. Surgical retractors were utilized to provide visualization of the vaginal canal, septum, and cervices. A physical examination confirmed a complete symmetric longitudinal septum, with a noted transection of the distal segment due to prior traumatic injury. Using forceps for traction, the 5-mm blunt-tip LigaSure device (Medtronic, Minneapolis, MN, USA) was employed to sequentially cauterize and divide the septum, starting distally and progressing toward the cervix. The anterior and posterior portions were addressed separately, followed by a division of the apical septum between the cervices, allowing for the complete removal of the longitudinal vaginal septum. Hemostasis was achieved with minimal blood loss, estimated at 5 mL. The surgical outcome was a single vaginal canal with two visible cervices (Figure 1). The patient tolerated the procedure well, without complications, and was discharged home the same day.

From the surgical procedure, multiple mucosa-covered soft tissue fragments were obtained and submitted for histopathologic evaluation. On gross inspection, no distinct masses were identified. Pathologic analysis revealed normal vaginal mucosa with no significant histopathologic changes.

**Figure 2:**
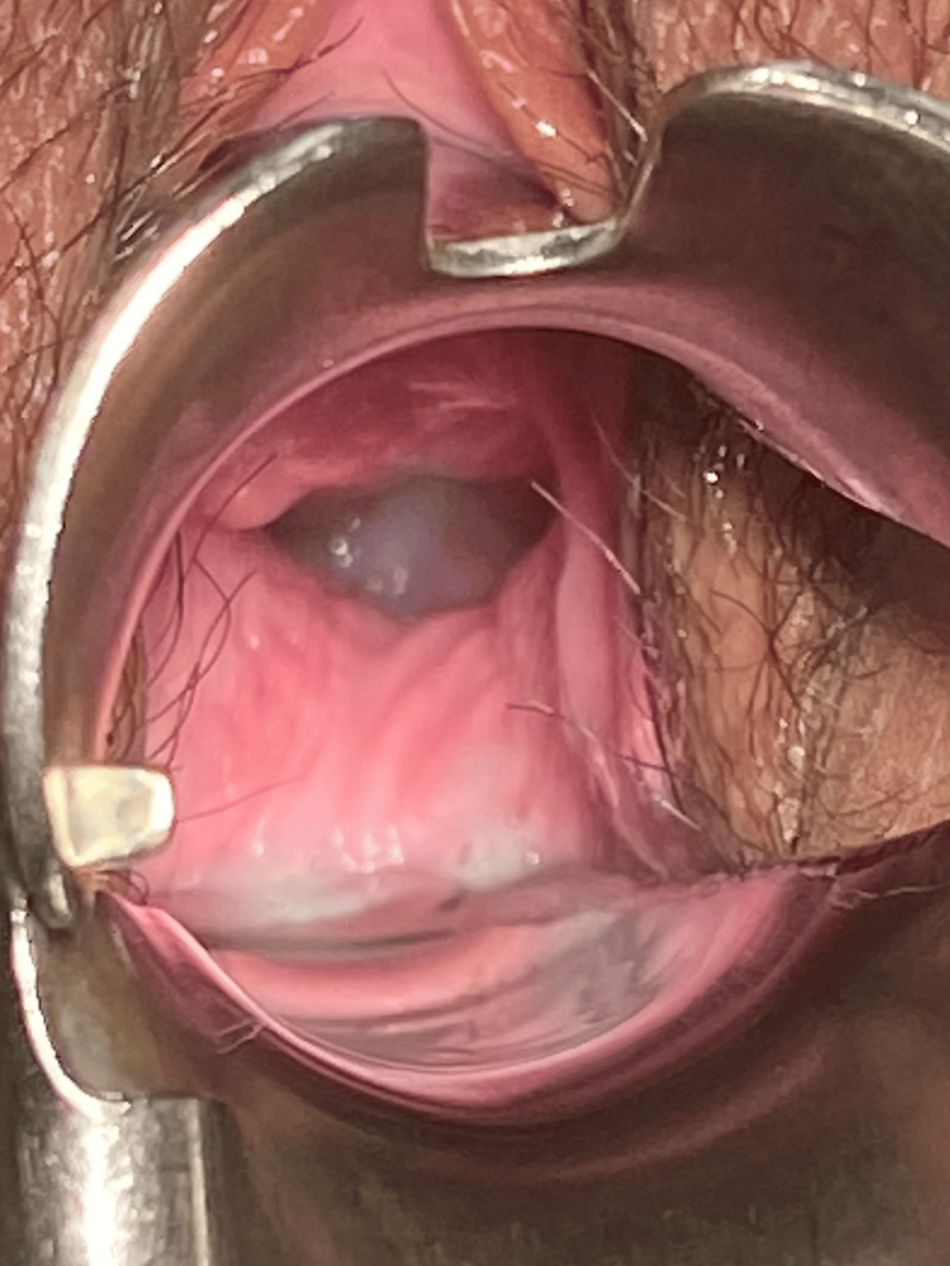
Postoperative vaginal anatomy demonstrating successful resection of a longitudinal vaginal septum

The patient missed her two-week post-operation appointment and was unable to present to the clinic until her six-week post-operation follow-up visit. She reported no pain or complications after longitudinal vaginal septum surgical excision. Notably, the patient reported the ability to engage in pain-free intercourse, which was the primary indication for surgical intervention. A physical exam revealed a single, well-healed, patent vaginal canal with no evidence of fistulas or other adverse complications (Figure 2). The overall surgical outcome was excellent.

## Discussion

The surgical management of longitudinal vaginal septa encompasses several documented techniques. In the traditional approach, gentle traction is applied to the septum using clamps or tissue forceps, and the line of attachment to the vaginal mucosa is carefully resected with scissors, both anteriorly and posteriorly. Subsequently, the incisions in the vaginal wall are closed with interrupted absorbable sutures to create a single vaginal canal [3]. However, this traditional method is associated with heavy bleeding, creating a cause for concern [4].

More recent advancements include hysteroscopic resection, which is a minimally invasive option that can be particularly beneficial for hymen preservation in young women [5,6]. Hysteroscopic resection is a safe alternative to conventional methods, as it provides good visualization, continuous irrigation, and a sutureless approach [5]. However, this method may only be performed easily in a specific case, such as when a longitudinal vaginal septum has caused an obstructed hemivagina with a large hematocolpos [6]. While hymen preservation can be a culturally relevant reason to pursue a hysteroscopic resection, this was not an indication for surgery in our patient.

The utilization of surgical innovations like staplers and Ligasure devices has also been documented for vaginal septum transection [7]. Gastrointestinal anastomosis (GIA) and endoscopic gastrointestinal anastomosis (EndoGIA) stapler devices are commonly used in gastrointestinal surgery, while the LigaSure device has been used in gynecological surgery. Both allow for the simultaneous resection of the vaginal septum and hemostasis; however, the use of staples may lead to irritation during intercourse in the future, if they are not adequately covered by epithelization [7].

Our experience highlights the potential advantages of the LigaSure device for vaginal septum resection, offering a safe and efficient approach compared to traditional methods. Building upon its established role in gynecological and intra-abdominal procedures, the LigaSure's mechanism of combined mechanical pressure and coagulation allowed for simultaneous dissection and cauterization of the septum [8]. Furthermore, the instrument's compact design provided ease of manipulation within the small and limited surgical field when working in the vaginal canal [8]. While some literature describes significant postoperative bleeding from the surgical site associated with the use of a LigaSure device, this complication was not observed in our patient [8]. In our experience, the LigaSure device enabled a successful resection of the vaginal septum with minimal blood loss and without technical difficulty.

## Conclusions

In summary, surgical correction of longitudinal vaginal septum provides a solution for reproductive challenges and the relief of symptoms such as dyspareunia, infertility, and dysmenorrhea. Although a great variety of surgical techniques exist for excising a longitudinal vaginal septum, the LigaSure device presents an efficient and safe option. Its combined dissection and hemostatic capabilities, along with its manageable size for precise manipulation, make it a valuable tool for this surgical treatment. However, careful attention to adjacent anatomical tissues and structures remains crucial to prevent complications, such as thermal injury or bleeding. Given the relative rarity of this condition, further comparative research on various surgical approaches and their subsequent outcomes is warranted. Furthermore, treatment should be patient-centered, with the appropriate surgical approach tailored to the individual patient’s clinical needs and preferences. The LigaSure device represents a promising alternative and should be considered a valuable tool in the surgical management of longitudinal vaginal septa.
